# AIMP3 depletion causes genome instability and loss of stemness in mouse embryonic stem cells

**DOI:** 10.1038/s41419-018-1037-4

**Published:** 2018-09-24

**Authors:** Sun Mi Kim, Yoon Jeon, Doyeun Kim, Hyonchol Jang, June Sung Bae, Mi Kyung Park, Hongtae Kim, Sunghoon Kim, Ho Lee

**Affiliations:** 1 Graduate School of Cancer Science and Policy, Research Institute, National Cancer Center, Gyeonggi, 10408 Republic of Korea; 2 Research Institute, National Cancer Center, Gyeonggi, 10408 Republic of Korea; 3 Medicinal Bioconvergence Research Center, Department of Pharmacology, Seoul National University, Seoul, 08826 Republic of Korea; 4 Department of Biological Science, Sungkyunkwan University, Suwon, 16419 Republic of Korea

## Abstract

Aminoacyl-tRNA synthetase-interacting multifunctional protein-3 (AIMP3) is a component of the multi-aminoacyl-tRNA synthetase complex and is involved in diverse cellular processes. Given that AIMP3 deficiency causes early embryonic lethality in mice, AIMP3 is expected to play a critical role in early mouse development. To elucidate a functional role of AIMP3 in early mouse development, we induced AIMP3 depletion in mouse embryonic stem cells (mESCs) derived from blastocysts of *AIMP3*^*f/f*^*; Cre*^*ERT2*^ mice. In the present study, AIMP3 depletion resulted in loss of self-renewal and ability to differentiate to three germ layers in mESCs. AIMP3 depletion led to accumulation of DNA damage by blocking double-strand break repair, in particular homologous recombination. Through microarray analysis, the p53 signaling pathway was identified as being activated in AIMP3-depleted mESCs. Knockdown of p53 rescued loss of stem cell characteristics by AIMP3 depletion in mESCs. These results imply that AIMP3 depletion in mESCs leads to accumulation of DNA damage and p53 transactivation, resulting in loss of stemness. We propose that AIMP3 is involved in maintenance of genome stability and stemness in mESCs.

## Introduction

The aminoacyl-tRNA synthetase-interacting multifunctional protein-3 (AIMP3)/p18 is involved in initiating mammalian translation through specific interaction with methionyl-tRNA synthetase^1,2^. In previous studies, AIMP3 was shown to play a role in diverse biological processes, such as response to DNA damage, oncogenic stress, and aging. Park et al. reported that AIMP3 mediates ataxia telangiectasia mutated (ATM)/ATM and RAD3-related (ATR)-dependent activation of p53 following DNA damage in cancer cells^3^. In addition, AIMP3 overexpression causes aging phenotypes in mice through downregulation of lamin A and cellular senescence in human mesenchymal stem cells^4,5^. Homozygous disruption of *AIMP3* gene in mice causes early embryonic lethality before embryonic day 8.5 (E8.5)^3^, implying that AIMP3 plays a critical role during early mouse embryo development^6,7^. However, a functional role for AIMP3 in early mouse embryonic development has not yet been identified.

Embryonic stem cells (ESCs) are derived from the inner cell mass of a blastocyst at embryonic day 3.5^8,9^. The main characteristic features of ESCs are self-renewal, which is the ability to continually generate new progeny cells identical to mother cells and pluripotency, which is the ability to differentiate into all cell lineages in the body^8,10^. Based on these features, ESCs are considered an attractive model system for studying early development^10–12^. In previous reports, cellular stresses, including DNA damage, oxidative stress or endoplasmic reticulum (ER) stress, were identified as affecting the self-renewal and pluripotency of mouse ESCs (mESCs)^13–16^. Although the leukemia inhibitory factor (LIF) signaling pathway and core transcription factors, such as OCT4, NANOG, and SOX2, are known to play critical roles in maintaining self-renewal and pluripotency in mESCs, other pluripotency regulatory factors have been recently described, indicating that self-renewal and pluripotency are regulated by a variety of complicated mechanisms^8,17^.

The tumor-suppressor p53 is known to regulate the transcription of genes involved in multiple cellular functions, including DNA repair, proliferation, apoptosis, and senescence, in response to genotoxic or cellular stresses^18,19^. Previous studies have demonstrated that p53 plays a critical role in mESCs differentiation and somatic cell reprogramming. DNA damage causes differentiation of mESCs in a p53-dependent manner^20^. DNA damage-induced p53 activation suppresses the transcription of key pluripotency factors, including *Oct4*, *Nanog*, and *Sox2*, and induces the transcription of differentiation-related factors^20,21^. In addition, p53 activation interferes with reprogramming somatic cells to induced pluripotent stem cells (iPSCs)^22,23^.

Herein, we demonstrated that AIMP3 depletion causes genomic instability and loss of stem cells characteristics including self-renewal and differentiation potential through p53 activation in mESCs. In mESCs, p53 knockdown rescued impaired stemness caused by AIMP3 depletion. Furthermore, AIMP3 depletion reduces the reprogramming efficiency of mouse embryonic fibroblasts (MEFs) to iPSCs. These data indicate that AIMP3 plays a role in the maintenance of genome stability and stem cell properties in mESCs.

## Results

### AIMP3 is involved in the maintenance of stemness in mESCs

**Fig. 1:**
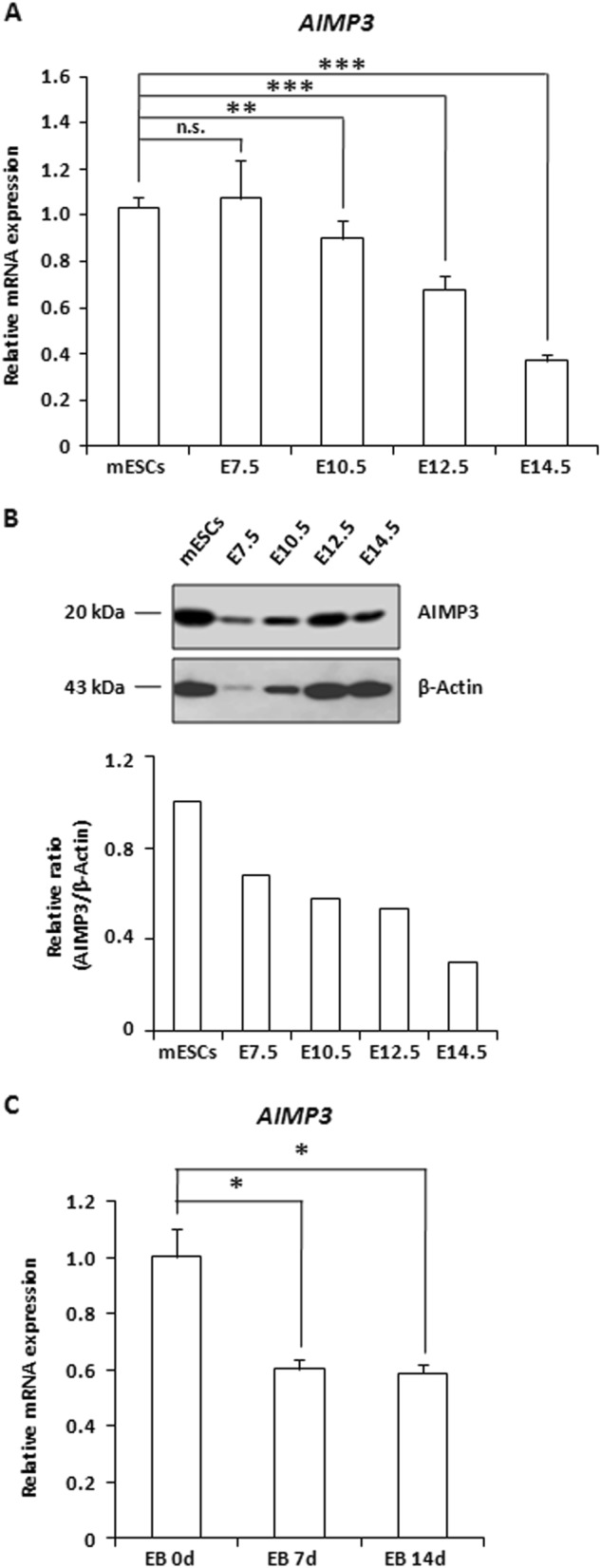
AIMP3 expression levels are decreased during development. **a**, **b** In mESCs and mouse embryos at different developmental stages, relative AIMP3 expression levels were assessed by qRT-PCR and western blot, respectively. E7.5 embryonic day 7.5, E10.5 embryonic day 10.5, E12.5 embryonic day 12.5, E14.5 embryonic day 14.5. **c** AIMP3 expression levels at the indicated times were determined by qRT-PCR during EB formation. Three independent experiments were performed for qRT-PCR, and results are expressed as the mean ± SD. *p *<* 0.05; ** *p* < 0.01; ***p < 0.001; n.s., not significant (*p* > 0.05)

It was previously reported that murine AIMP3 deficiency showed early embryonic lethality^3^. We found that AIMP3-depleted embryos were resorbed at the preimplantation stage and that AIMP3-deficient embryonic cells showed defect in outgrowth (Fig. S1). Given that AIMP3 could be a critical factor in early mouse embryonic development, AIMP3 was expected to exhibit a temporal gene expression pattern during embryonic development. AIMP3 expression levels were high in ES cells and gradually decreased at the mRNA and protein levels during development (Fig. 1a, b). Consistent with these results, *AIMP3* expression levels were reduced throughout embryoid body (EB) formation, mimicking postimplantation embryo development (Fig. 1c). These data indicate that AIMP3 has a critical function in the early stages of mouse embryonic development.

To investigate whether AIMP3 has critical functions in early embryonic development, mESC clones were derived from blastocysts of *AIMP3*^*f/f*^*; Cre*^*ERT2*^ mice (Fig. S2A, S2B and S2C). Cre^ERT2^ enables regulation of target gene expression using tamoxifen treatment. In *AIMP3*^*f/f*^*;Cre*^*ERT2*^ ESCs, tamoxifen treatment successfully led to AIMP3 depletion in dose- and time-dependent manners (Fig. S2D and S2E).

AIMP3 depletion in mESCs did not affects expression levels of preimplantation (*Nr0b1* and *Dnmt3l*) and postimplantation epiblast markers (*Fgf5*, *Otx2*, and *Oct6*) (data not shown). However, upon transition from naive pluripotent state (culture condition with 2i + LIF) to postimplantation (with withdrawal of 2i + LIF), AIMP3-depleted mESCs showed increased expression of preimplantation epiblast markers and decreased expression of postimplantation epiblast markers compared with the control (Fig. S3). These data suggest that AIMP3 depletion causes peri-implantation lethality between E4.5 and E5.5 in mice.

**Fig. 2:**
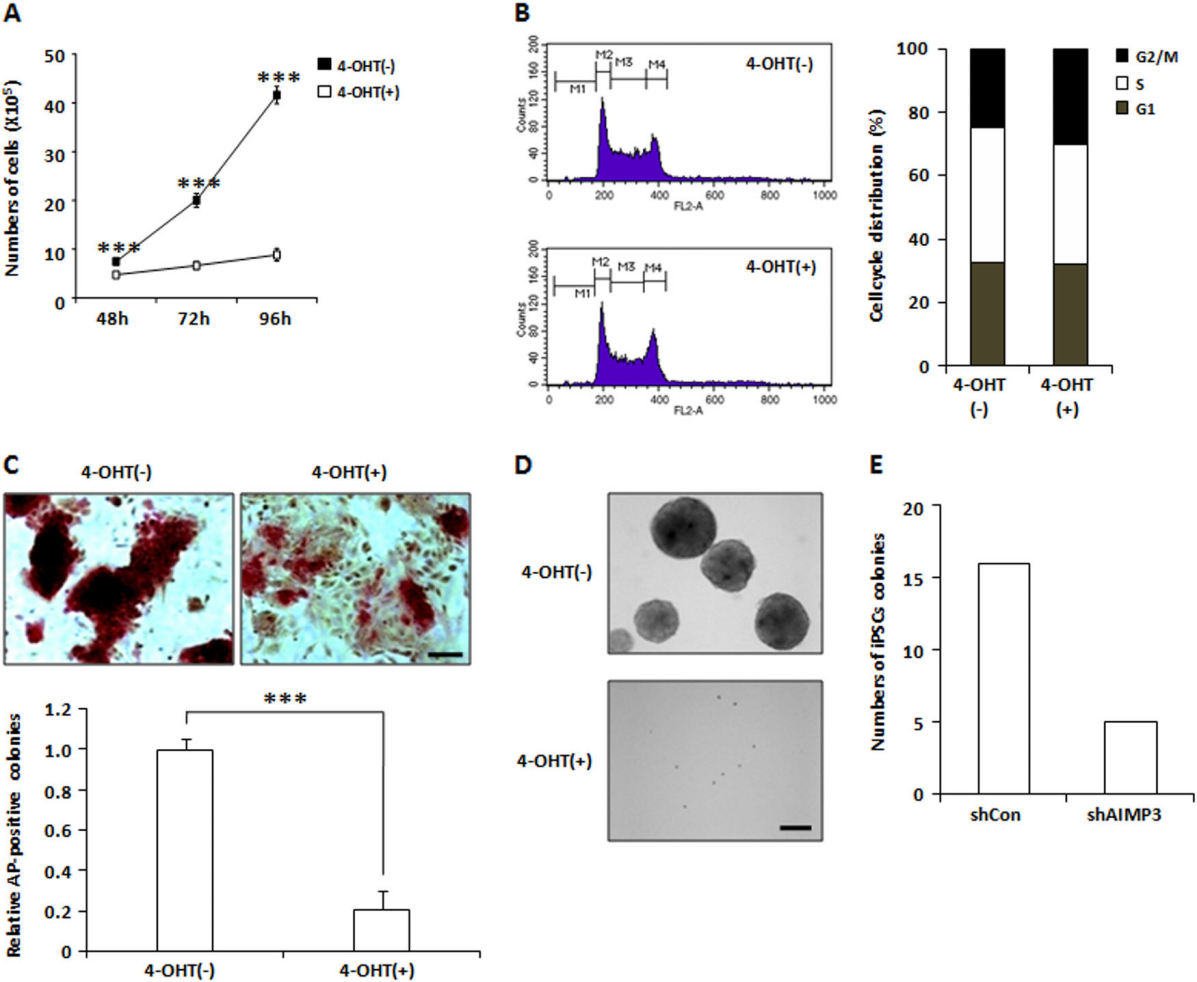
AIMP3 deficiency results in loss of stem cell characteristics in mESCs. **a** *AIMP3*^*f/f*^*; Cre*^*ERT2*^ mESCs were treated with or without 2 μM 4-OHT. At the indicated time points, cells were harvested by trypsinization and counted. Three independent experiments were performed, and results are expressed as the mean ± SD. ***p < 0.001. **b** Cells were treated with or without 2 μM 4-OHT for 3 days. Cell cycle distribution was assessed by PI staining and FACS analysis. **c** Cells were treated with or without 2 μM 4-OHT for 3 days and stained with AP. The upper panel shows representative images from AP staining (magnification ×20), and the lower panel shows the ratio of AP-positive stained colonies between samples treated with and without 4-OHT. Three independent experiments were performed, and results are expressed as the mean ± SD. ***p < 0.001. **d** Cells were treated with or without 2 μM 4-OHT for 4 days. Representative images show the results of EB formation using the hanging drop method. Magnification ×10. **e** After reprogramming of Oct4-GFP MEFs, reprogrammed iPSCs colonies were detected by AP staining and GFP expression. AP/GFP double-positive colonies were counted. Left panel, lentiviral shRNA-mediated AIMP3 knockdown was identified by western blot analysis. Right panel, the number of AP/GFP double-positive iPSC colonies

We first investigated whether AIMP3 loss affects stem cell characteristics including self-renewal and differentiation potential of mESCs. The growth rate of AIMP3-depleted mESCs was dramatically reduced compared with the control, and loss of AIMP3 induced cell cycle arrest at the G2/M phase in mESCs (Fig. 2a, b). AIMP3 depletion-induced G2/M arrest was confirmed by western blot analysis in which key factors for G2/M transition, such as Cyclin B1, CDC25C, and CDK1, were downregulated in AIMP3-depleted cells (Fig. S4A). In addition, AIMP3 depletion was shown to slightly induce cell death based on flow cytometry after annexin-V/propidium iodide (PI) double staining (Fig. S4B). Depletion of AIMP3 resulted in differentiated cell morphology and reduction of alkaline phosphatase (AP) activity, which is indicative of stemness (Fig. 2c). AIMP3 depletion also blocked EB formation from mESCs (Fig. 2d). These data suggest that AIMP3 depletion causes loss of self-renewal and stemness in mESCs.

Factors involved in maintaining the stemness of ES cells have been reported to influence generation of iPSCs^24^. AIMP3 expression was shown to be upregulated after 8 days of OSKM (OCT4, SOX2, KLF4, and MYC) induction in MEF cells during iPSCs formation^25^. Therefore, we investigated whether AIMP3 affects the reprogramming efficiency of MEF to iPSCs. Lentiviruses expressing short hairpin RNA (shRNA) targeting *AIMP3* were generated, and knockdown of AIMP3 by lentiviral shRNA was verified (Fig. S5). Reprogramming efficiency of MEF to iPSCs in AIMP3-depleted cells was reduced to 30% compared with the control (Fig. 2e).

**Fig. 3:**
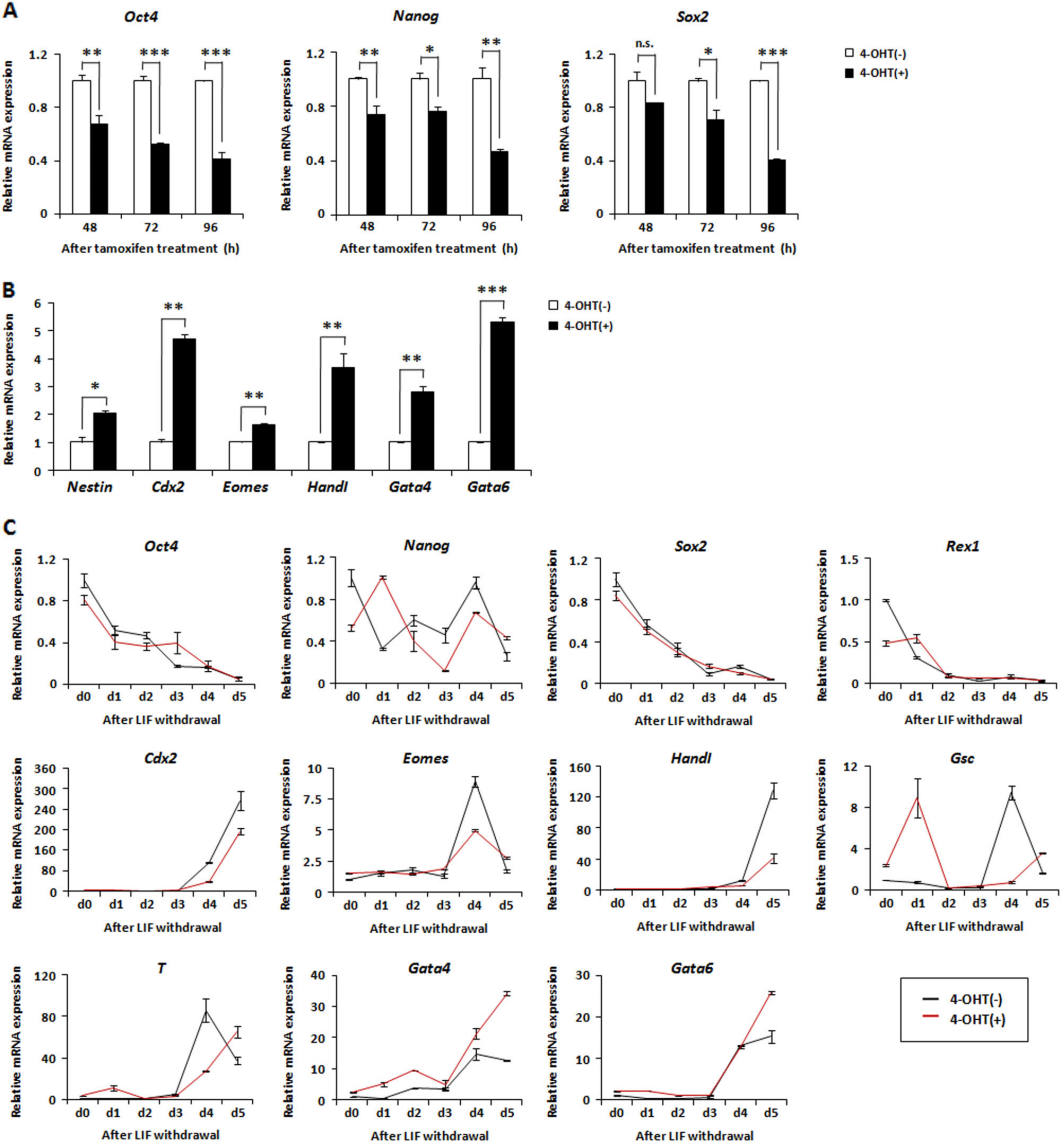
Loss of AIMP3 affects expression of pluripotency- and differentiation-related factors in mESCs. **a**, **b** Cells were incubated with or without 2 μM 4-OHT for 3 days and harvested. mRNA expression of indicated markers was measured by qRT-PCR. Results are expressed as a ratio of each marker to the control. Three independent experiments were performed, and results from qRT-PCR are expressed as the mean ± SD. *p < 0.05; **p < 0.01; ***p *<* 0.001; n.s., not significant (*p* > 0.05) . **c** Cells were incubated with or without 2 μM 4-OHT for 2 days in the presence of LIF. After LIF withdrawal, cells were harvested at the indicated times. Then, expression of the indicated markers was measured by qRT-PCR. The *Y* axis represents the ratio of expression of each marker to the control (at day 0 without 4-OHT)

Consistent with reduced AP staining in AIMP3-deficient mESCs, expression levels of the pluripotency factors, *Oct4*, *Nanog*, *Sox2, Rex1*, and *SSEA1* were downregulated by AIMP3 depletion (Fig. 3a and Fig. S6). In contrast to the reduction in pluripotency markers, AIMP3 depletion caused an increase in the expression of differentiation-related markers, including ectoderm (*Nestin*), trophectoderm (*Cdx2* and *Eomes*), mesoderm (*HandI*), and endoderm (*Gata4* and *Gata6*) (Fig. 3b). Changes in the expression of pluripotency and differentiation-related factors by AIMP3 depletion were confirmed in experiments with small interfering RNA (siRNA)-mediated knockdown of AIMP3 (Fig. S7). Additionally, AIMP3 overexpression rescued the effects of AIMP3 depletion on expression of pluripotency or differentiation-related markers in mESCs, indicating specific regulation of those factors by AIMP3 (Fig. S8).

Next, we investigated whether AIMP3 depletion impairs differentiation potential of mESCs into three germ layers through in vitro and in vivo differentiation assays. In vitro differentiation assay through LIF withdrawal showed that trophectoderm markers (*Cdx2* and *Eomes*) and mesoderm markers (*HandI* and *T*) were not fully activated in AIMP-depleted mESCs, compared with control cells. Another mesoderm marker (*Gsc*) was abnormally activated at earlier time point compared with the control. Endoderm markers (*Gata4 and Gata6*) were more activated in AIMP3-depleted mESCs compared with the control (Fig. 3c). Consistent with these results, AIMP3 depletion attenuated teratoma formation from mESCs (Fig. S9). Although tetratoma with three germ layers was normally generated in all mice injected with control mESCs, only two of five mice injected with AIMP3-depleted mESCs formed teratoma that was smaller in size than the control and only showed mesodermal tissue such as cartilages. Taken together, these results suggest that loss of AIMP3 impairs the differentiation potential of mESCs.

### Depletion of AIMP3 induces p53 transactivation in mESCs

**Fig. 4:**
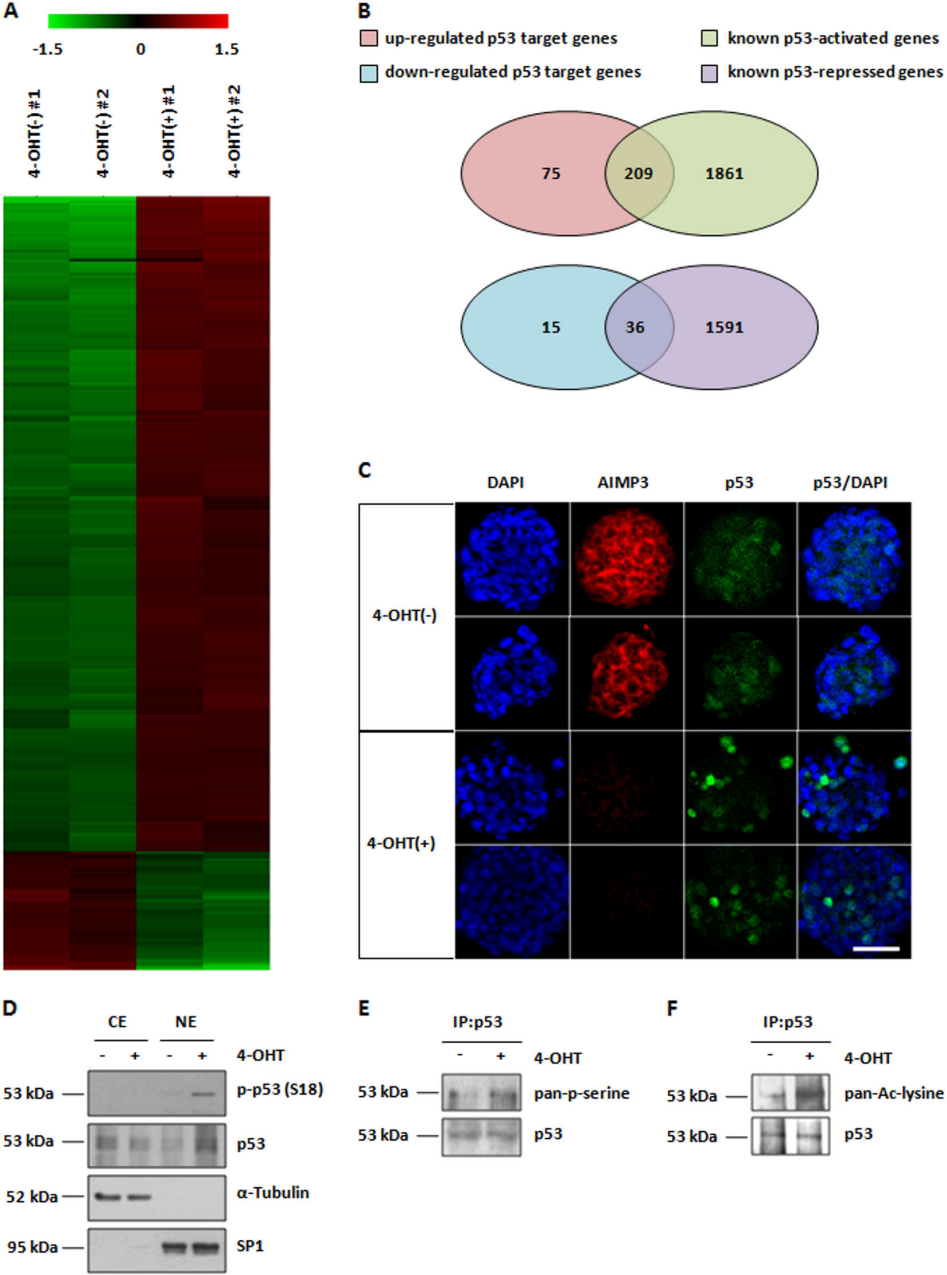
Loss of AIMP3 induces transactivation of p53 in mESCs. **a** Hierarchical cluster analysis was performed on 333 p53 target genes that were altered >1.5-fold, with a *p*-value of < 0.05 between mESCs treated with and without 2 μM 4-OHT. Red squares represent upregulation and green squares represent downregulation. **b** A Venn diagram illustrates the overlap between p53 target genes upregulated or downregulated by AIMP3 depletion as compared with known p53-activated or -repressed genes. **c** Cells were treated with or without 2 μM 4-OHT for 2 days and stained as indicated. Representative images are shown. Magnification ×20. **d** Cells were treated with or without 2 μM 4-OHT for 2 days. Indicated proteins were identified from cytoplasmic extract (CE) and nuclear extract (NE) by western blot analysis. α-Tubulin and SP1 were used as markers of the cytoplasmic and nuclear fractions, respectively. **e**, **f** After treatment with or without 2 μM 4-OHT for 2 days, cells were harvested and lysed for IP

To elucidate the regulatory mechanisms of AIMP3 regarding ability to proliferate and differentiate into three germ layers in mESCs, we performed microarray analysis in control and AIMP3-depleted mESCs. Compared with the control, 1070 genes had significant changes ( >1.5-fold, *p* < 0.05) in expression in AIMP3-depleted mESCs. Among these genes, 880 genes were upregulated and 190 genes were downregulated. Through analysis with DAVID bioinformatics online tools, the p53 signaling pathway was found to be activated in AIMP3-depleted cells (Fig. S10). In 2012, Li and colleagues identified 3697 genes that were transcriptional targets of p53 in mESCs^20^. They showed that p53 activates 2070 genes and represses 1627 genes. To identify alterations in p53 target genes from our microarray data, we integrated the p53 target genes reported by Li with genes from our microarray data that had significant changes. This analysis revealed that approximately 31% (333 genes) of the 1070 genes, which showed significant changes based on our microarray data, are involved with p53 target genes (Fig. 4a). Among 333 genes, about 74% (209 genes) of activated genes (284 genes) and about 71% (36 genes) of repressed genes (51 genes) were matched with p53-activated and -repressed genes, which are reported by Li, respectively (Fig. 4b). Using quantitative reverse transcription PCR (qRT-PCR), we identified that well-known p53 target genes are transcriptionally activated in AIMP3-depleted mESCs (Fig. S11). In addition, immunofluorescent staining and western blot analysis revealed nuclear translocation of p53 by AIMP3 depletion in mESCs (Fig. 4c, d). Western blot analysis of immunoprecipitated p53 showed that phosphorylation and acetylation of p53, which are critical events for p53 activation^26^, were increased in AIMP3-depleted mESCs (Fig. 4e, f). These data suggest that depletion of AIMP3 results in transcriptional activation of p53 in mESCs.

### Depletion of AIMP3 leads to DNA damage in mESCs

**Fig. 5:**
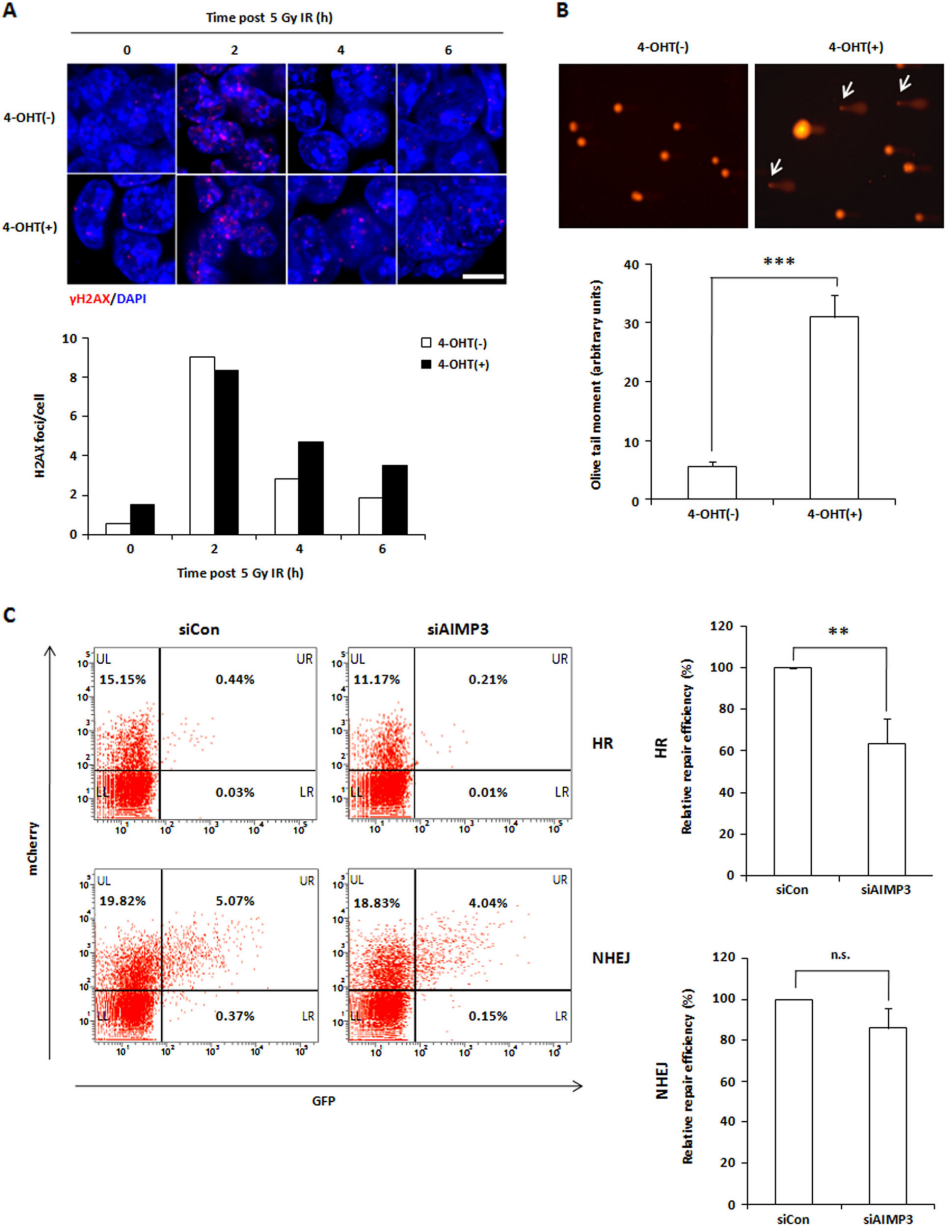
Loss of AIMP3 accumulates DNA damage through impairment of DSB repair in mESCs. **a** Cells were incubated with or without 2 μM 4-OHT for 2 days and exposed to 5 Gy IR for the indicated time points. Cells were then stained as indicated. The upper panel indicates representative images (magnification ×40), and the lower panel is a graph or relative γH2AX foci counts per cell calculated using ImageJ software. Two independent experiments were performed for this assay. **b** Cells were incubated with or without 2 μM 4-OHT for 4 days and harvested for the comet assay. The upper panel indicates representative images obtained from 20 to 25 randomly selected images. White arrows indicate DNA comet tails. The lower panel is a graph of relative olive tail moment calculated using the OpenComet plug-in (v.13) in ImageJ software. Three independent experiments were performed, and results are expressed as the mean ± SEM. ***p *<* 0.001. **c** After 24 h post-transfection of control siRNA (siCon) or *AIMP3-*targeting siRNA (siAIMP3), U2OS cells were transfected with linearized DR-GFP (for the HR assay) or EJ5-GFP (for the NHEJ assay) and mCherry plasmids to monitor transfection efficiency. After 3 days of second transfection, the percentages of GFP- or mCherry-positive cells was determined by flow cytometry. Then, HR or NHEJ repair efficiency for each sample was determined by normalizing the percentage of GFP-positive cells to the percentage of mCherry-positive cells. The left panel represents representative images from flow cytometry. The *X* and *Y* axes indicate GFP-positive and mCherry-positive cells, respectively. The graph on the right panel represents relative HR or NHEJ repair efficiency normalized to the control. Three independent experiments were performed and results are represented as the mean ± SD. **p < 0.01

In mESCs, DNA-damaged cells are removed from the pluripotent pool through apoptosis or differentiation to prevent propagation of genomic abnormalities to daughter cells cells^9,27–29^. Previous reports have shown that induction of DNA damage signals increased p53 activity in mESCs^20,30^. Given that AIMP3 is known to be important for maintaining chromosomal stability in somatic cells^3,31^, we hypothesized that AIMP3 depletion-induced genomic instability is involved in p53-dependent impairment of stemness in mESCs. To examine this hypothesis, we investigated whether AIMP3 depletion affects DNA damage response in mESCs. γH2AX foci levels in AIMP3-depleted mESCs were higher than in normal cells without any stimulus (Fig. 5a). Increased olive tail moment in comet assays was also observed in AIMP3-depleted mESCs (Fig. 5b). These results indicate that AIMP3 depletion leads to an accumulation of DNA double-strand breaks (DSBs) in mESCs.

In general, ATM and ATR are primarily activated after recognition of DSBs, and γH2AX foci are detected at the site of DSBs in response to DNA damage, thus activating DNA damage signaling^32^. AIMP3 has been reported to translocate to the nucleus and activate ATM and ATR upon DNA damage in somatic cells^3^. Therefore, we tested whether DNA damage induces activation of ATM and ATR in AIMP3-depleted mESCs. As shown in Fig. S12 and S13, phosphorylation and foci formation of ATM and ATR in AIMP3-depleted mESCs were similar to control cells following exposure to 5 Gy irradiation (IR). Furthermore, at 2 h after 5 Gy IR, γH2AX foci formation in mutant cells was similar compared with control cells (Fig. 5a). These data demonstrate that AIMP3 depletion has little effect on recognition of DSBs in mESCs.

We next investigated if AIMP3 depletion affects downstream repair events. After 5 Gy IR, the rate of γH2AX foci clearance was markedly slower in AIMP3-depleted mESCs compared with the control (Fig. 5a). Although there was little change at 2 h, the number of γH2AX foci per cell in AIMP3-depleted mESCs was 1.7-fold and 1.9-fold higher compared with the control at 4 h and 6 h after IR, respectively. These results suggest that AIMP3 depletion impairs DNA repair processes in mESCs. Homologous recombination (HR) and non-homologous end joining (NHEJ) are two major repair pathways of DSBs^33^. It is generally accepted that DSBs in somatic cells are repaired by NHEJ pathway and that mESCs predominantly depend on HR to repair DSBs^34^. To further investigate whether AIMP3 depletion affects DSBs repair, we performed the established HR and NHEJ reporter assays in U2OS cells^35^. For HR and NHEJ reporter assays, cells were transfected with linearized DR-GFP and EJ5-GFP constructs, respectively. The DR-GFP construct contains a modified GFP gene with an I-SceI recognition site and an internal GFP template for HR repair. The EJ5-GFP contruct contains a promoter that is seperated from a GFP gene by a puromycin gene flanked by two I-SceI sites. Because linearized constructs harbor site-specific DSBs within the mutant GFP allele by I-SceI restriction enzyme, GFP is normally expressed if HR or NHEJ repair is occurred in transfected cells. Therefore, GFP expression can be used an indicator of DSBs repair. HR efficiency in AIMP3-depleted cells was reduced to 60% compared with the control (Fig. 5c). Results of the NHEJ reporter assay in U2OS cells showed that the efficiency of NHEJ repair was not significantly affected by AIMP3 knockdown. These data suggest that AIMP3 depletion causes impaired DSBs repair, mainly HR in mESCs, resulting in accumulation of DNA damage.

### Loss of stemness in AIMP3-depleted mESCs is p53 dependent

**Fig. 6:**
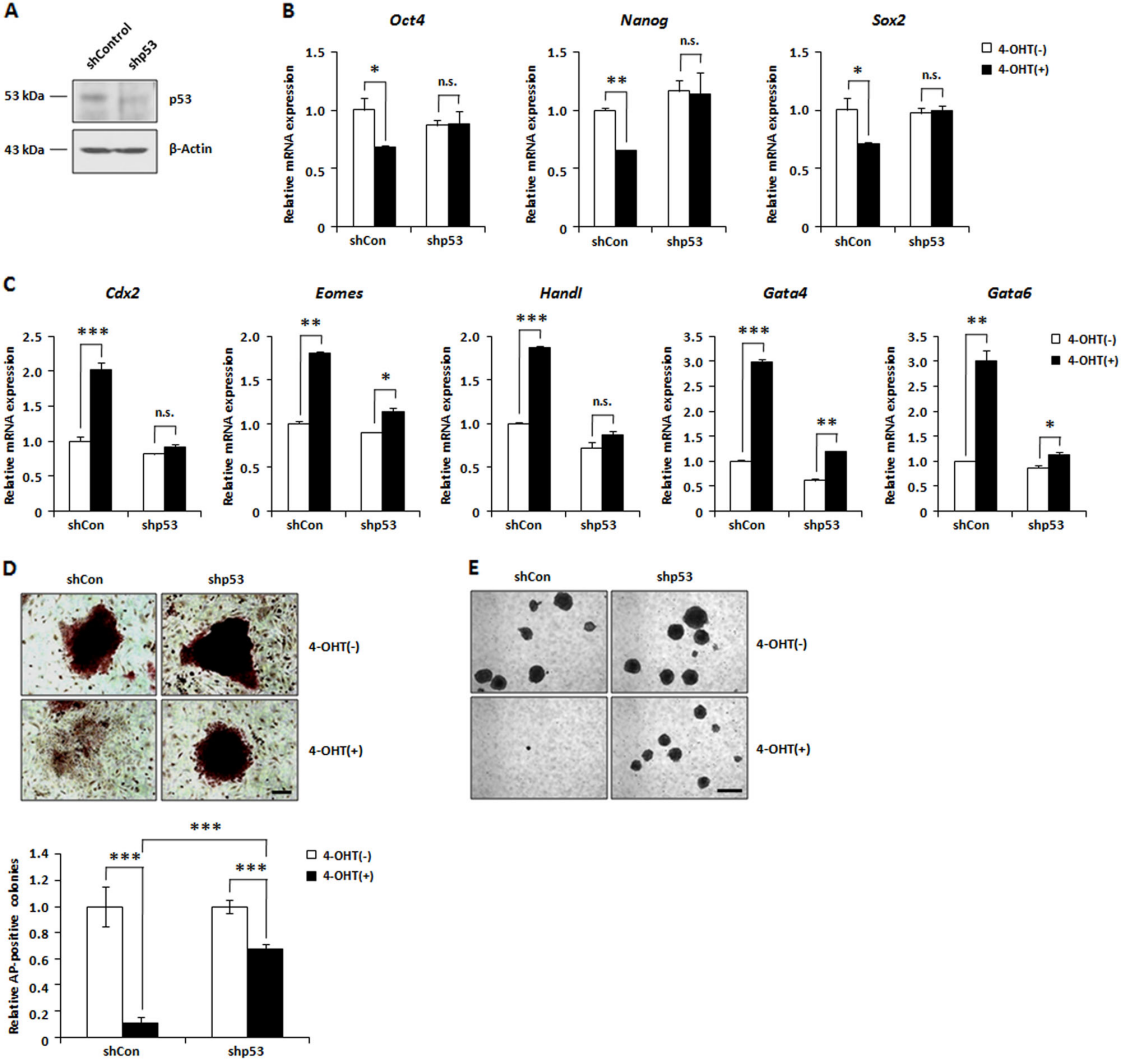
p53 is involved in loss of stemness in AIMP3-depleted mESCs. **a** After generating p53 stable knockdown mESCs by transduction of *p53*-specific shRNA, p53 depletion was examined by western blot analysis. **b**, **c** After incubation with or without 2 μM 4-OHT for 2 days in control shRNA-transduced or *p53* shRNA-transduced mESCs, relative expression levels of the indicated markers were analyzed by qRT-PCR. **d** After treatment with or without 2 μM 4-OHT for 3 days, cells were stained with AP. The left panel indicates representative images. Magnification ×10. The right panel is a graph of the relative ratio of AP-positive stained colonies. Three independent experiments were performed for qRT-PCR and AP staining, and results are expressed as the mean ± SD. *p < 0.05; **p < 0.01; ***p < 0.001; n.s., not significant (*p* > 0.05) . **e** Cells were incubated with or without 2 μM 4-OHT for 5 days. Representative images indicate the results of EB formation. Magnification ×5

Activation of p53 plays a key role in promoting differentiation of mESCs^21,36,37^. We investigated whether p53 activation is involved in impairment of a self-renewing pluripotent state in AIMP3-depleted mESCs. For p53 knockdown, *AIMP3*^*f/f*^*; Cre*^*ERT2*^ mESCs were transduced with a control or lentivirus containing a shRNA sequence directed against p53. Knockdown of p53 was confirmed by western blot analysis (Fig. 6a). Expression of shRNA targeting p53 completely reversed the reduced expression levels of *Oct4*, *Nanog*, and *Sox2* by AIMP3 depletion (Fig. 6b). The increased expression of differentiation-related markers by AIMP3 depletion was also rescued upon p53 knockdown (Fig. 6c). In addition, AIMP3 depletion did not cause loss of ES cell morphology and AP staining in p53 shRNA-transduced mESCs (Fig. 6d). Consistent with these results, inhibition of EB formation by AIMP3 loss was not observed following p53 knockdown (Fig. 6e). In vivo differentiation assay using teratoma formation showed that p53 knockdown rescued the failure of teratoma formation caused by AIMP3 depletion (Fig. S9). These results indicate that loss of self-renewing pluripotent state in AIMP3-depleted mESCs is mainly mediated by p53 .

p53 is known to be phosphorylated and activated by several kinases in DNA damage responses. In mammalian cells, three members of the phosphatidylinositol-3-OH-kinase like family of protein kinases, including ATM, ATR, and DNA-dependent protein kinase (DNA-PK), act as initiators DNA damage responses and activate p53 upon DNA damage^38^. Because AIMP3 depletion itself did not activate ATM and ATR, we investigated whether DNA-PK plays a role in p53 activation in AIMP3-deficient mESCs. As shown in Fig. S14, increased phosphorylation of p53 by AIMP3 loss was blocked in the presence of Nu7026, an inhibitor of DNA-PK, suggesting that p53 activation in AIMP3-deficient mESCs may occur in a DNA-PK-dependent manner.

## Discussion

**Fig. 7:**
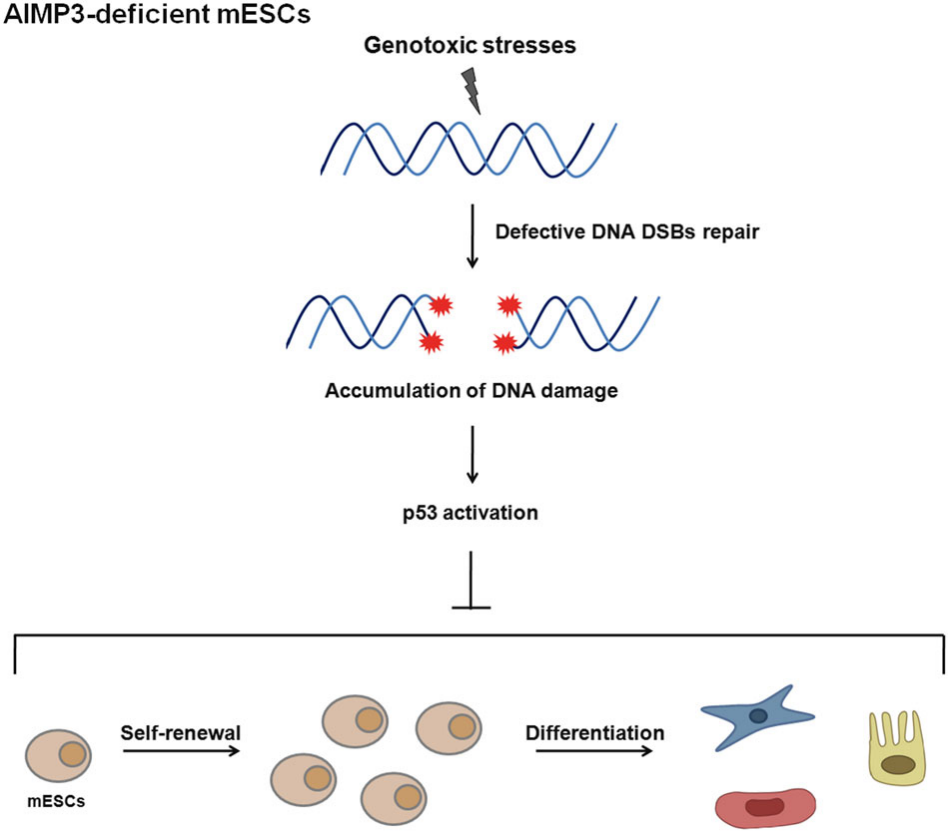
An illustration of the role of AIMP3 in maintaining genome stability and stemness of mESCs. Under genotoxic stresses including replication stress or ionizing radiation, AIMP3 deficiency accumulates DNA damage through blocking DNA double-strand breaks repair, mainly homologous recombination, and impairs the maintenance of stemness in mESCs

In the present study, we showed that AIMP3 depletion causes genomic instability by impairing DNA repair processes in mESCs. In addition, AIMP3 depletion in mESCs caused loss of stem cell characteristics in a p53-dependent manner (Fig. 7). This is the first report demonstrating that AIMP3 has a role in the maintenance of stemness in mESCs. These findings are supported by data showing embryonic lethality by AIMP3 deficiency and high AIMP3 expression levels in mESCs and at early stages of embryo development.

Even if AIMP3-depleted mESCs showed features of loss of pluripotency such as reduced AP staining, reduced expression of pluripotency markers, and impaired teratoma formation, it is unclear whether AIMP3 is a critical factor for regulation of pluripotency. In this study, AIMP3 depletion inhibited cell cycle progression and led to cell death in mESCs. We cannot exclude the possibility that these features of loss of pluripotency by AIMP3 depletion are a secondary phenomenon caused by impaired cell growth of mESCs.

AIMP3 depletion resulted in an increased olive tail moment in the comet assay and delayed clearance of γH2AX foci in mESCs. Delayed clearance of γH2AX by AIMP3 depletion was also observed in MEFs (data not shown). HR and NHEJ efficiency analysis showed that AIMP3 depletion leads to accumulated DNA damage through defects in DNA repair pathways, mainly HR. ESCs show an active DNA damage response to prevent propagation of mutations caused by genotoxic stresses, such as rapid proliferation-associated replication stress to daughter cells^27^. mESCs are predominantly dependent on HR to repair DSBs and show high expression of HR-related proteins. During differentiation, levels of DNA ligase IV, a rate-limiting factor for NHEJ, are increased, and ES cells utilize NHEJ rather than HR to repair DSBs^39^. Therefore, HR activity is necessary for genome integrity and stemness maintenance in mESCs. Although the regulatory mechanism involving AIMP3 in HR repair needs further investigation, it is important to note that AIMP3 is involved in maintaining genome integrity through regulation of HR in mESCs.

Previous studies have shown that p53 plays a negative role in regulating ESC pluripotency and somatic cell reprogramming in mice^20–23,36,37^. The roles of p53 in mESCs are similar to those in human ESCs (hESCs). p53 promotes differentiation of hESCs by suppressing expression of pluripotency-related genes^40^. Reprogramming efficiency of human somatic cells is increased through inhibition of p53 activity^41,42^. Moreover, downregulation of p53 increased the reprogramming efficiency of goat embryonic fibroblasts to iPSCs^43^. Collectively, these results suggest that p53 could be a common barrier to somatic cell reprogramming in multiple species. The present study revealed that AIMP3 depletion causes p53 transactivation in mESCs and decreases reprogramming efficiency during iPSCs formation from MEFs, implying that p53 activation caused by AIMP3 depletion blocks the processes of reprogramming of MEFs to iPSCs. Further studies will be needed to demonstrate how AIMP3 plays a critical role during the reprogramming in various species.

Although we identified that AIMP3 depletion causes transcriptional activation of p53, previous studies have shown that AIMP3 is a positive regulator for p53 activation via ATM in somatic or cancer cells^3,44^. This functional discrepancy can be explained by different cellular contexts. Previous studies have shown the effects of AIMP3 on p53 activation in somatic cells, such as transformed MEF and cancer cells, but not in mESCs. mESCs have their own unique gene expression profiles^45^. Differences in gene expression between different cell types may lead to alterations in protein–protein interactions and cellular localization, resulting in different protein functions depending on cell type. Exposure to genotoxic stress has been reported to induce nuclear translocation of AIMP3 in somatic cells^3,46^. However, nuclear translocalization of AIMP3 after IR was not observed in mESCs (Fig. S15). We also determined that p53 phosphorylation by exposure to 5 Gy of IR is inhibited in AIMP3-depleted MEF cells, but not in AIMP3-depleted mESCs (Fig. S12). These results support our hypothesis that the function of AIMP3 depends on the cellular context.

This study shows that p53 activation may be mediated in a DNA-PK-dependent manner in AIMP3-depleted mESCs. It was previously reported that DNA-PK plays diverse roles as a tumor suppressor and a transcriptional regulator independent of DNA repair^47^. Meanwhile, DNA-PK functionally complements ATM and is involved in HR repair^47,48^. Additional studies are required to determine whether and how AIMP3 depletion activates DNA-PK followed by p53 activation and loss of stemness in mESCs.

In conclusion, these results show that AIMP3 depletion causes accumulation of DNA damage due to impairment of DNA repair processes and loss of stem cell characteristics through p53 activation in mESCs. These findings provide supportive evidence to explain embryonic lethality in AIMP3-deficient mice and describe the role of AIMP3 in maintenance of genome instability and stemness in mESCs.

## Materials and methods

### Cell culture

mESCs were cultured as previously described^49^. For incubation with drugs, siRNA and lentiviral particles, mESCs were cultured in MEF-conditioned medium. U2OS cells were maintained in Dulbecco’s modified Eagle’s medium (DMEM) supplemented with 10% fetal bovine serum (FBS) and 5000 units/ml penicillin/streptomycin. All cells were maintained in a humidified incubator at 37 ℃ with 5% CO_2_.

### Extraction of total RNA and protein from mouse embryo

Whole embryos from C57BL/6 mice were homogenized in TRIzol reagent (Ambion, Austin, TX, USA) for extraction of total RNA or RIPA lysis buffer (GenDEPOT, Barker, TX, USA) for extraction of total protein using a TissueLyzer II (Qiagen, Hilden, Germany) according to the manufacturer’s manual.

### Antibodies and chemical

4-Hydroxytamoxifen (4-OHT) was purchased from Sigma-Aldrich (St Louis, MO, USA). Antibodies for western blotting were purchased from the following sources: anti-AIMP3 (sc-376019), anti-β-Actin (sc-47778), anti-p53 (sc-100), anti-α-Tubulin (sc-5286), anti-SP1 (sc-420), anti-phosphoserine (sc-81514), and anti-pan-acetyl (sc-8663-R) antibodies were obtained from Santa Cruz Biotechnology (Dallas, TX, USA). Anti-phospho p53 (S18, 9286) was purchased from Cell Signaling (Danvers, MA, USA). Antibodies for immnunofluorescence staining were purchased from the following sources: anti-p53 (sc-1312) and anti-AIMP3 antibodies (NMS-01–0002) were purchased from Santa Cruz Biotechnology and Neomics (Gyeonggi, Republic of Korea), respectively. Anti-γHAX antibody (07–164) was purchased from Millipore (Boston, MA, USA).

### Cell growth assay

Cells were seeded at a density of 1 × 10^5^ cells in a 24-well culture plate. After incubation with 4-OHT, cells were harvested by trypsinization and counted by 2% Trypan blue staining in a hemacytometer.

### Cell cycle analysis

Cells were fixed overnight in ice-cold 70% ethanol (EtOH) at 4 °C. Then, cells were washed with 1× phosphate-buffered saline (PBS) and incubated in 0.5 mg/ml RNase A solution (Sigma-Aldrich) at 37 °C. After incubation for 10 min, cells were stained with 50 μg/ml PI (Sigma-Aldrich) for 30 min. Cell cycle distribution was assessed by flow cytometry (BD Biosciences, San Diego, CA, USA).

### AP staining

Cells were fixed in ice-cold 100% methanol at room temperature (RT). After fixation, cells were stained with AP detection kit (Vector laboratories, Burlingame, CA, USA) for 30 min at RT. After washing with distilled water, AP-positive colonies were counted from scanned images using Image J software.

### EB formation

EB formation was performed according to a previous report^50^. mESCs were prepared at a concentration of 5 × 10^5^ cells in differentiation medium (DMEM supplemented with 10% FBS, 0.055 mM β-mertcaptoethanol, 2 mM l-glutamine and 5000 units/ml of penicillin/streptomycin). In all, 20 μl of the cell suspension was placed onto the lid of Petri dishes. After incubation for 2 days at 37 °C, EBs formed in drops were transferred into Petri dishes containing differentiation medium and further incubated for 2 days at 37 °C. For the attached EBs development, EBs were plated into six-well culture plates on day 4 and incubated at 37 °C.

### Lentiviral construction, production, and transduction

The shRNA targeting AIMP3 was cloned into lentiviral vector pLKO.1 (shAIMP3/pLKO). The cloned lentiviral vector was co-transfected with packaging vectors (psPAX2 and VSV-G) into 293FT cells. At 48 h post-transfection, supernatant containing lentiviral particles was collected, filtered through a 0.45 μm membrane and concentrated using Lenti-X concentrator (Clontech, Palo Alto, CA). shRNA sequences are listed in Supplementary Table 1. Control lentiviral particles and lentiviral particles containing shRNA to p53 were purchased from Santa Cruz Biotechnology. Cells were cultured with lentiviral particles in the presence of polybrene (5 μg/ml). At 48 h post-transduction, transduced cells were harvested for further studies or selected by 1 μg/ml puromycin (Sigma-Aldrich).

### Reprogramming

Reprogramming was performed as previously described^51^. Briefly, Oct4-GFP transgenic MEFs harboring Oct4-response element were prepared from pOct4-GFP mouse (Jackson Laboratory, Bar Harbor, Maine, USA). The Oct4-GFP MEFs were transduced with retroviruses encoding Oct4, Sox2, and Klf4 plus/minus lentiviruses encoding shRNA targeting *AIMP3* (shAIMP3/pLKO). After 24 h post-transduction, mitomycin C-inactivated feeder cells were added. After 21 days post-transduction, cells were stained with AP. To monitor the expression of endogenous Oct4, expression of GFP was detected using a fluorescence microscopy (Carl Zeiss, Jena, Germany). Finally, AP and GFP double-positive colonies were counted as the successfully reprogrammed iPSCs.

### qRT-PCR

qRT-PCR was performed as previously described^52^. Briefly, total RNA was extracted using TRIzol reagent (Ambion) and converted to complementary DNA using M-MLV reverse transcriptase (Promega, Madison, WI, USA) according to the manufacturer’s instructions. qRT-PCR was performed on the LightCycler®480 system (Roche, Mannheim, Germany) using the SYBR Green reagents (Takara, Tokyo, Japan). The expression levels of each mRNAs were normalized against GAPDH. The primers used for qRT-PCR are listed in Supplementary Table 2.

### siRNA transfection

U2OS cells were transfected with 30 μmol/L of siRNA with INTERFERin® siRNA/miRNA transfection reagent (Polyplus transfection, Illkirch, France). Next day, cells were transfected with plasmids for the DSBs repair assay. siRNAs against human *AIMP3* are listed in Supplementary Table 3.

### Western blot analysis

Cells were lysed with RIPA lysis buffer (GenDEPOT). After protein quantification using Bradford protein assay reagents (Bio-Rad, Hercules, CA, USA), equal amounts of protein were loaded into the well of the sodium dodecyl sulfate-polyacrylamide gel electrophoresis (SDS-PAGE) gel and fractionated. After transfer onto a nitrocellulose membrane (Bio-Rad), membranes were blocked with 5% bovine serum albumin (BSA) in PBS + 0.1% Tween 20 (0.1% PBST) for 1 h at RT. After incubation with primary antibodies at 4 °C overnight, membranes were incubated with horseradish peroxidase (HRP)-conjugated secondary antibodies. The blot was developed by ECL chemiluminescence detection system (Abfrontier, Seoul, Republic of Korea).

### Microarray and DAVID bioinformatics analysis

Microarray experiments were performed as previously described using a commercial microarray service from eBiogen^22^. Briefly, total RNA was extracted using TRIzol reagent. Amplification and labeling were performed using Low RNA Input Linear Amplification kit PLUS (Agilent, Santa Clara, CA, USA). Amplified RNA was hybridized to Agilent Mouse GE 8 × 60K using Agilent’s Gene Expression Hybridization Kit. Arrays were scanned with DNA microarray scanner running Feature Extraction Software 10.7 (Agilent). Raw expression values were normalized using GeneSpringGX 7.3.1 software (Agilent). The averages of normalized ratios were calculated by dividing the average of control normalized signal intensity by the average of test normalized signal intensity. The data are accessible through GEO Series accession number GSE104241. The gene list, which is significantly up or downregulated by AIMP3 depletion, was submitted to Datatabase for Annotation, Visualization and Integrated Discovery (DAVID) functional annotation tool (version 6.7) and the lists of enriched Kyoto Encyclopedia of Genes and Genome (KEGG) pathways were obtained.

### Immunofluorescence staining

Cells were fixed in ice-cold 100% methanol and then washed with ice-cold 1× PBS. After permeabilization with 1× PBS containing 0.25% Triton X-100 for 10 min, cells were blocked with 3% BSA in 0.1% PBST for 1 h at RT. After incubation with primary antibodies at 4 °C overnight, cells were incubated with fluorescent dye-conjugated secondary antibodies for 1 h at RT. All primary antibodies were diluted 1:100 in blocking solution and all secondary antibodies were diluted 1:250 in blocking solution. Cells were mounted with mounting medium with 4,6-diamidino-2-phenylindole (DAPI; Vector laboratories). Immunofluorescence images were acquired using a confocal microscopy (LSM510; Carl Zeiss).

### γH2AX foci counting

The number of γH2AX foci and DAPI-stained nuclei in each image were determined using ImageJ software. γH2AX foci in unstained nuclei were not counted. The number of γH2AX foci was divided by the number of DAPI-stained nuclei to determine the average number of H2AX foci per cell. More than 200 DAPI-positive nuclei were counted for each sample.

### Immunoprecipitation

In total, 1 mg of cell lysate was precleared for 1 h with 20 μl of protein A/G agarose beads (50% slurry) containing 2 μg of normal goat IgG. After centrifugation, supernatants were incubated with 2 μg of goat anti-p53 antibody at 4 °C overnight. After incubation with 20 μl of 50% bead slurry for 4 h, beads were washed three times with RIPA lysis buffer. Immunocomplexes were eluted for 5 min at 95 °C with 2× SDS-PAGE sample buffer and subjected to western blot. For detection of immunoprecipitated p53, we used mouse anti-p53 antibody as a primary antibody.

### Comet assay

Comet assay was performed as previously described^53^. Briefly, cells were harvested and suspended in 1% low melt agarose (37 ℃) and 2 × 10^4^ cells were spread on a slide and treated with lysis buffer and alkaline solution. After electrophoresis, slides were stained with PI (1 μg/ml). More than 20 images per slide were randomly selected from each sample, and the olive tail moment was calculated by OpenComet plug-in (v.13) in ImageJ software.

### DNA DSBs repair assay

The HR or NHEJ-mediated DSBs repair assay was performed as previously described with some modifications^54^. Briefly, DR-GFP (for HR repair assay) and EJ5-GFP (for NHEJ repair assay) were linearized by I-SceI restriction enzyme and then purified using Labopass^TM^ gel extraction kit (Cosmo Genetech, Seoul, Republic of Korea). U2OS cells were transfected with linearized DR-GFP or EJ5-GFP plasmids using jetPEI® reagent (Polyplus transfection). We co-transfected mCherry plasmid to monitor transfection efficiency of each sample. After 3 days post-transfection, we identified the GFP-positive cells and mCherry-positive cells for each sample using flow cytometry (BD Biosciences).

### Statistical analysis

The Student’s *t*-test was conducted to assess statistical significance between groups, and *p* *<* 0.05 was considered to be statistically significant. Statistical significance is indicated by the asterisks.

## Electronic supplementary material

Supplementary data
