# Supplementary material for: AIMP3 depletion causes genome instability and loss of stemness in mouse embryonic stem cells
Source: Cell Death Dis. 2018 Sep 24;9(10):972. doi: 10.1038/s41419-018-1037-4 (PMC6155375; doi:10.1038/s41419-018-1037-4)
Supplement: Supplementary file 1 — Supplementary data [file 41419_2018_1037_MOESM1_ESM.docx]

**Supplementary Table 1.** List of shRNA target sequences.

| **Gene** | **Name** | **Sequence** |
| --- | --- | --- |
| *AIMP3* | shAIMP3-1 | 5’-CGGCCAGTATTGTCTTCATCAACTCGAGTTGATGAA  GACAATACTGGTTTTG-3’ |
|  |  | 5’-ATTCAAAAACCAGTATTGTCTTCATCAACTCGAGTT  GATGAAGACAATACTGG-3’ |
|  | shAIMP3-2 | 5’-CCGGGCAACATCTGTCCAGTATTCTCGAGAATACTG  GACAGATGTTGCTTTTG-3’ |
|  |  | 5’-AATTCAAAAAGCAACATCTGTCCAGTATTCTCGAGA  ATACTGGACAGATGTTGC-3’ |

**Supplementary Table 2**. List of primer sequences for qRT-PCR

| **Gene** | **Primer sequence** |
| --- | --- |
| *Oct4* | sense 5’-TTGGGCTAGAGAAGGATGTGGTT-3’  antisense 5’-GGAAAAGGGACTGAGAGAGTGTGG-3’ |
| *Nanog* | sense 5’-AACCTTTTCAGAAATCCCTTCC-3’  antisense 5’-GAGGCAGGTCTTCAGAGGAA-3’ |
| *Sox2* | sense 5’-GCACATGAACGGCTGGAGCAACG-3’  antisense 5’-TGCTGCGAGTAGGACATGCTGTAGG-3’ |
| *Rex1* | sense 5’-CCAGGTTCTGGAAGCGAGTT-3’  antisense 5’-AGTAATGAGCTCGCCCCAAC-3’ |
| *SSEA1* | sense 5’-TGGTGAAGGAACTCCACGAC-3’  antisense 5’-CTCCAGGGCTTTGCCAGTTA-3’ |
| *Cdx2* | sense 5’-AGCAGTCCCTAGGAAGCCAA-3’  antisense 5’-TCTCGGAGAGCCCAAGTGT-3’ |
| *Eomes* | sense 5’-CCACCGCCCACTACAATGTT-3’  antisense 5’-CCCAAAGGAAATCTCCTGCCT-3’ |
| *Nestin* | sense 5’-GCTGGAACAGAGATTGGAAGG-3’  antisense 5’-CCAGGATCTGAGCGATCTGAC-3’ |
| *HandI* | sense 5’-TCGCCTACTTGATGGACGTG-3’  antisense 5’-GGCCTGGTCTCACTGGTTTA-3’ |
| *Gata4* | sense 5’-CCTGGAAGACACCCCAATCTC-3’  antisense 5’-AGGTAGTGTCCCGTCCCATCT-3’ |
| *Gata6* | sense 5’-GCTGAACGGAACGTACCACC-3’  antisense 5’-ACAGTGGCGTCTGGATGGAG-3’ |
| *Nr0b1* | sense 5’-GGTCCAGGCCATCAAGAGTT-3’  antisense 5’-CCAGGCAGGTCTGGGTTAAA-3’ |
| *Dnmt3l* | sense 5’-TGTGGAGCAACATTCCAGGG-3’  antisense 5’-CAGGAGGTCAACTTTCGGGG0-3’ |
| *Fgf5* | sense 5’-ACCCGGATGGCAAAGTCAAT-3’  antisense 5’-TTCAGTTCTGTGGATCGCGG-3’ |
| *Otx2* | sense 5’-GCGAAGGGAGAGGACGACATTT-3’  antisense 5’-CTGCTGTTGGCGGCACTTAG-3’ |
| *Oct6* | sense 5’-AGTTCGCCAAGCAGTTCAAG-3’  antisense 5’-TGGTCTGCGAGAACACGTTA-3’ |
| *p53* | sense 5’-CCATGGCCCCTGTCATCTTT-3’  antisense 5’-TGAGGGGAGGAGAGTACGTG-3’ |
| *p21* | sense 5’-TTGTCGCTGTCTTGCACTCT-3’  antisense 5’-AGACCAATCTGCGCTTGGAG-3’ |
| *Mdm2* | sense 5’-AGTCAGCAAGACTCTGGCAC-3’  antisense 5’-AGGTTTCTCTTCTGGTGGCG-3’ |
| *14-3-3* | sense 5’-TGTGGCGAAGACTAGGAGGA-3’  antisense 5’-GTCTCGAGAGTAACGCTGGG-3’ |
| *Gapdh* | sense 5’-TGTCGTGGAGTCTACTGGTGTC-3’  antisense 5’-GCTAAGCAGTTGGTGGTGCAGG-3’ |

**Supplementary Table 3.** List of siRNA target sequences.

| **Gene** | **Name** | **Target sequence** |
| --- | --- | --- |
| mouse *AIMP3* | siAIMP3-1 | 5’-CCAGUAUUGUCUUCAUCAATT-3’  5’-UUGAUGAAGACAAUACUGGTT-3’ |
|  | siAIMP3-2 | 5’-GCAACAUCUGUCCAGUAUUTT-3’  5’-AAUACUGGACAGAUGUUGCTT-3’ |
| human *AIMP3* | siAIMP3 | 5’-CCAAGUCUAACAGGAUUGACUACUATT-3’  5’-UAGUAGUCAAUCCUGUUAGACUUGGTT-3’ |

**Supplementary figure 1**

**Supplementary figure 2**

**Supplementary figure 3**

**Supplementary figure 4**

**Supplementary figure 5**

**Supplementary figure 6**

**Supplementary figure 7**

**Supplementary figure 8**

**Supplementary figure 9**

**Supplementary figure 10**


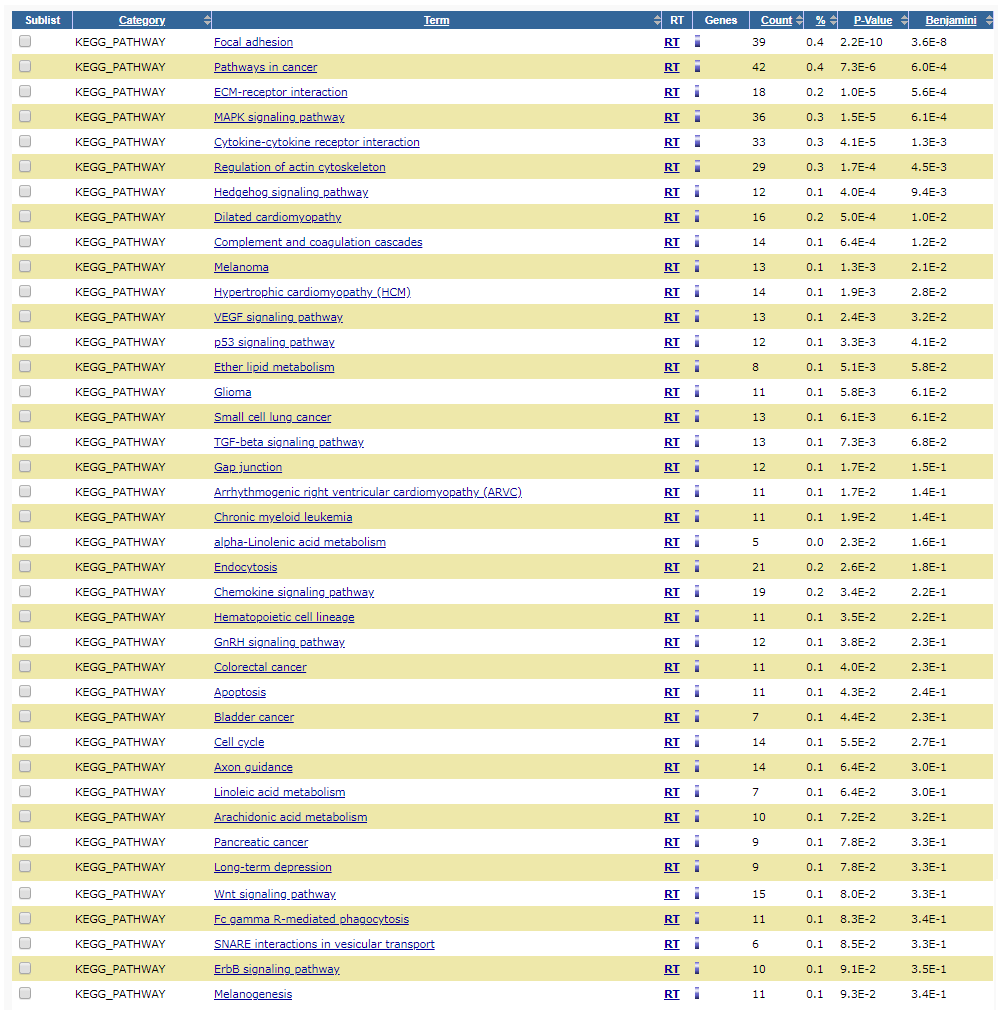


**Supplementary figure 11**

**Supplementary figure 12**

**Supplementary figure 13**

**Supplementary figure 14**

**Supplementary figure 15**

**SUPPLEMENTARY FIGURE LEGENDS**

**Supplementary Figure 1.** *AIMP3*^-/-^ embryos show defective development in mice. (A) *AIMP3*^-/-^ embryo, but not the *AIMP3*^+/+^ embryo, was resorbed at E6.5. (B) E3.5 embryos were collected and cultured *in vitro* for 3 days. *AIMP3*^+/+^ embryos, but not *AIMP3*^-/-^ embryos, are composed of an inner cell mass surrounded by trophoblast giant cells. Magnifications are indicated at the top of each image.

**Supplementary Figure 2.** Targeting the murine *AIMP3* locus. (A) Schematic representation of the *AIMP3* locus, the targeting vector and the targeted loci are shown. Exon 1-4, southern blot probe, translation start site (ATG), puromycin cassette (puro), diphtheria toxin A chain gene (DT-A), FRT sequence (F), *loxP* sequence (triangle) and restriction sites (Bh, *BamH*I; Hp, *Hpa*I; S, *Sal*I) are shown. (B) Southern blot analysis of a representative *AIMP3^+/puro^* ES cell clone is shown. For *Hpa*I digestion, bands representing wild-type and mutant alleles are ~9.0 kb and ~11.0 kb, respectively. (C) PCR analysis with genomic DNA extracted from ES cell clones. The circle number represents the PCR reaction described in (A). (D) mESCs were incubated with or without 2 μM 4-OHT for 2 days and harvested for Western blot. The graph depicts the relative expression of AIMP3. (E) mESCs were incubated with or without 2 μM 4-OHT for indicated times and harvested for qRT-PCR. Three independent experiments were performed for qRT-PCR, and results are expressed as the mean ± SD. *** *p* < 0.001.

**Supplementary Figure 3**. Effects of AIMP3 depletion on the expression levels of epiblast markers. mESCs were maintained in 2i (3 μM CHIR99021+1 μM PD035901) + LIF medium. After treatment of 2 μM 4-OHT for 2 days, mESCs were maintained without 2i+LIF for 48 h. Cells were harvested for qRT-PCR. Three independent experiments were performed and results are represented as the mean ± SD. *** *p* < 0.001.

**Supplementary Figure 4**. Analysis of cell cycle and cell death by AIMP3 depletion. (A) After tamoxifen treatment for 2 days, cells were harvested for Western blot analysis. (B) After tamoxifen treatment for 2 days, cells were stained with annexin V and propidium iodide and analyzed through flow cytometry. Annexin V^+^/PI^-^, early apoptotic cells; Annexin V^+^/PI^+^, late apoptotic and necrotic cells; Annexin V^-^/PI^+^, necrotic cells.

**Supplementary Figure 5**. Lentiviral shRNA-mediated AIMP3 knockdown. After 2 days post-transduction with lentiviruses targeting AIMP3, mESCs were harvested for Western blot analaysis. Upper image represents a result of Western blot analysis and lower graph indicates the relative expression of AIMP3 protein.

**Supplementary Figure 6**. AIMP3 depletion causes reduction of the expression of *Rex1* and *SSEA1* mRNA. Cells were incubated with or without 2 μM 4-OHT for 3 days and harvested. The expression of *Rex1* and *SSEA1*mRNA was measured by qRT-PCR. Results are represented as a ratio of the expression of each marker to the control. Three independent experiments were performed and results of qRT-PCR are expressed as the mean ± SD. * *p*< 0.05; ** *p*< 0.01; *** *p* < 0.001.

**Supplementary Figure 7**. AIMP3 knockdown affects expression of pluripotency- and differentiation-related factors in mESCs. (A) and (B) After transfection with control or *AIMP3* targeting siRNA for 3 days, cells were harvested for Western blot analysis and qRT-PCR. Three independent experiments were performed and results from qRT-PCR are represented as the mean ± SD. * *p* < 0.05; ** *p* < 0.01; *** *p* < 0.001 (n.s, not significant, *p* > 0.05).

**Supplementary Figure 8.** AIMP3 overexpression rescues the effects of tamoxifen-induced AIMP3 deficiency on the expression of pluripotency or differentiation-related genes. After 2 days post-transfection with a flag-AIMP3 expression vector, cells were treated with or without 2 μM 4-OHT for 2 days and harvested for Western blot and qRT-PCR. (A) Endogenous and flag-tagged AIMP3 were detected using anti-AIMP3 or anti-flag antibody, respectively. Red and black arrows on AIMP3 blot indicate the flag-tagged and endogenous AIMP3, respectively. (B) Three independent experiments were performed for qRT-PCR and results are represented as the mean ± SD. * *p* < 0.05; ** *p* < 0.01; *** *p* < 0.001 (n.s, not significant, *p* > 0.05).

**Supplementary Figure 9**. AIMP3 depletion in mESCs impairs pluripotency to differentiate three germ layers. In control and p53-depleted mESCs, AIMP3 depletion was induced by treatment of 2 μM 4-OHT for 2 days. These cells were subcutaneously injected into nude mice (n=5 per group). After 4 weeks, teratomas were harvested for histological analysis. (A) Whole images of teratoma isolated from each mouse. (B) Representative images of hematoxylin and eosin (H&E) staining for teratoma isolated from each group. The region of mesoderm, ectoderm and endoderm are marked by dashed line. NF; not found. Magnification 20X. Scale bar, 100 μm.

**Supplementary Figure 10**. The list of enriched Kyoto Encyclopedia of Genes and Genome (KEGG) pathways analyzed through DAVID bioinformatics tool in AIMP3-depleted mESCs. The gene list, which is significantly altered by AIMP3 depletion, was submitted to DAVID functional annotation tool. The table shows the list of enriched KEGG pathways obtained from DAVID bioinformatics analysis.

**Supplementary Figure 11.** *AIMP3* depletion increases expression of p53 target genes in mESCs. Cells were treated with or without 2 μM 4-OHT and harvested after 3 days for qRT-PCR analysis. Three independent experiments were performed for qRT-PCR, and results are expressed as the mean ± SD. * *p* < 0.05; ** *p* < 0.01; *** *p* < 0.001 (n.s, not significant, *p* > 0.05).

**Supplementary Figure 12.** DNA damage response in *AIMP3*-depleted mESCs and MEF. Cells were exposed to 5 Gy γ-irradiation (IR) and harvested at the indicated time points for Western blot analysis.

**Supplementary Figure 13**. Foci formation of phospho-ATM in control and AIMP3-depleted cells on exposure to IR. Cells were incubated with or without 2 μM 4-OHT for 2 days and exposed to 5 Gy IR for 1 h. These cells were stained as described in Materials and Methods.

**Supplementary Figure 14**. Increased phosphorylation of p53 by AIMP3 was blocked in the presence of Nu7026, an inhibitor of DNA-PK. Cells were treated with or without 2 μM 4-OHT for 2 days in the presence of Nu7026 and then harvested for Western blot analysis. Magnification 40X. Scale bar, 10 μm.

**Supplementary Figure 15.** AIMP3 does not translocate to the nucleus under exposure to γ-irradiation in mESCs. mESCs were exposed to 5 Gy γ-irradiation and harvested at the indicated time points for immunofluorescence staining. Representative images indicate staining for γH2AX and AIMP3 obtained from confocal microscopy. Magnification 40X. Scale bar, 10 μm.

**SUPPLEMENTARY MATERIALS AND METHODS**

**Histological Analysis**

On E6.5 day, the embryos and placentas were fixed and embedded in paraffin. For histological analysis, 4-μm sections were stained with hematoxylin and eosin according to the manufacturer’s protocol. Hematoxylin and eosin were purchased from Sigma-Aldrich (St Louis, MO, USA).

**Blastocyst outgrowth assay**

Blastocyst outgrowth assay was performed as previously described^1^. Briefly, blastocysts were collected on E3.5 and cultured in Dulbecco’s modified Eagle medium (DMEM; Welgene Inc., Daegu, Republic of Korea) containing 15% fetal bovine serum (FBS; Hyclone, Logan, UT, USA), 0.055 mM β-mertcaptoethanol (Gibco, Grand Usland, NY, USA), 2 mM L-glutamine (Gibco), 0.1 mM nonessential amino acid (Gibco) without leukemia inhibitory factor. The cultured embryos were photographed daily and harvested for genotyping on day 5.

**Murine *AIMP3* gene-targeting**

A 15-kb DNA fragment containing exon 1, 2, 3 and 4 of the murine *AIMP3* gene was retrieved from BAC clones (bMQ-308J16) into a pBluescript phagemid system to a previously reported procedure^2^. Generation of targeted ES cell clones and germline transmission of the *AIMP3*^puro^ allele were performed as previously described^1^. All mice strains were backcrossed more than 6 generations to C57BL/6. This study was reviewed and approved by the Institutional Animal Care and Use Committee (IACUC) of National Cancer Center Research Institute.

**Cloning of flag-tagged AIMP3 expression vector and transient transfection**

Flag-AIMP3/pcDNA3.1 was constructed by inserting mouse *AIMP3* cDNA into the pcDNA3.1-Flag vector. For overexpression of Flag-AIMP3 in mESCs, mixtures of 2X10^6^ cells and 2 μg plasmid DNA were loaded on the Neon pipette (Invitrogen, Carlsbad, CA, USA) and transfected using the electroporation parameters, 1,250 voltage/20 ms/2 pulse.

**Preparation of MEF**

MEFs were generated from embryonic day 14.5 (E14.5) embryos according to a previous report^1^. MEFs were maintained in DMEM (Welgene Inc.) supplemented with 10% FBS, 0.055 mM β-mercaptoethanol, 2mM L-glutamine and 1% penicillin/streptomycin (Gibco) in a humidified incubator with 5% CO_2_.

**Maintenance of naive ground state in mESCs and induction of postimplantation epiblast state**

To maintain naïve ground state, mESCs were maintained in Dulbecco’s modified Eagle medium (DMEM, Welgene Inc.) supplemented with 15% fetal bovine serum (FBS; Hyclone), 0.055 mM β-mertcaptoethanol (Gibco), 2 mM L-glutamine (Gibco), 0.1 mM nonessential amino acid (Gibco), 5,000 units/ml of penicillin/streptomycin (Gibco), 3 μM CHIR99021 (Sigma-Aldrich, St Louis, MO, USA), 1 μM PD035901 (Selleckchem, Houston, TX, USA) and 1,000 units/ml leukemia inhibitory factor (LIF) (Millipore, Darmstadt, Germany). After incubation with or without 2μM tamoxifen for 48 h, cells were maintained growth medium without 2i (CHIR99021 and PD035901)+LIF for 48 h to induce postimplantation epiblast state.

**Reagent and antibodies**

Antibodies for Western blotting were obtained from the following sources: Anti-β-Actin (sc-47778), anti-Cyclin B1 (sc-245), anti-CDC25C (sc-13138), anti-CDK1 (sc-53219), anti-CDK4 (sc-23896), anti-Cyclin D1 (sc-8396), anti-Cyclin E (sc-377100) and anti-flag (sc-166355) antibodies were obtained from Santa Cruz Biotechnology (Dallas, TX, USA). Anti-phospho p53 (S18, 9286) and anti-phospho ATR (S428, 2853) antibodies were purchased from Cell Signaling (Danvers, MA, USA). For immunofluorescence staining, anti-γH2AX (05-636) antibody was purchased from Millipore (Bedford, MA, USA). For Western blotting and immunofluorescence staining, anti-phospho ATM (S1981, 05-740) and anti-AIMP3 (NMS-01-0002) antibodies were purchased from Millipore and Neomics (Gyeonggi, Republic of Korea), respectively.

**Transfection of AIMP3 siRNA**

Transient siRNA transfection of mESCs was performed using the Neon transfection system (Invitrogen, Carlsbad, CA, USA). Mixtures of 2 X 10^6^ cells and 100 pmole siRNA against AIMP3 were loaded on the Neon pipette and transfected using the electroporation parameters, 1,250 voltage / 20 ms / 2 pulse. siRNAs against mouse *AIMP3* are listed in Supplementary Table 3.

**Teratoma formation**

Nude mice were purchased from Jackson Laboratories (Bar Harbor, ME, USA). This study was reviewed and approved by the Institutional Animal Care and Use Committee (IACUC) of National Cancer Center Research Institute. 1 X 10^6^ cells were mixed with 100 μl matrigel (BD biosciences, Bedford, MA, USA) and injected subcutaneously into 6 weeks-old nude mice. At 4 weeks postinjection, teratomas were harvested. After fixation with 10% neutral buffered formalin, teratomas were embedded in paraffin and analyzed through hematoxylin and eosin staining. **SUPPLEMENTARY REFERENCES**

1. Jeon, Y. et al. TopBP1 deficiency causes an early embryonic lethality and induces cellular senescence in primary cells. *J. Biol. Chem.* **286**, 5414-5422 (2011).
2. Warming, S. et al. Simple and highly efficient BAC recombineering using galK selection. *Nucleic Acids Res.* **33,** e36 (2005).
